# ASYMMETRIC LEAVES1 regulates abscission zone placement in *Arabidopsis* flowers

**DOI:** 10.1186/s12870-014-0195-5

**Published:** 2014-07-20

**Authors:** Catherine M Gubert, Megan E Christy, Denise L Ward, William D Groner, Sarah J Liljegren

**Affiliations:** 1 Department of Biology, University of Mississippi, Oxford 38677, MS, USA

**Keywords:** Abscission, Floral organ shedding, Organ boundary, Flower development, AS1, BP

## Abstract

**Background:**

The sepals, petals and stamens of *Arabidopsis* flowers detach via abscission zones formed at their boundaries with the underlying receptacle. The ASYMMETRIC LEAVES1 (AS1) MYB transcription factor plays a critical role in setting boundaries between newly formed leaf primordia and the shoot meristem. By repressing expression of a set of *KNOTTED1-LIKE HOMEODOMAIN* (*KNOX*) genes from developing leaf primordia, AS1 and its partner ASYMMETRIC LEAVES2 allow the patterning and differentiation of leaves to proceed. Here we show a unique role for AS1 in establishing the positions of the sepal and petal abscission zones in *Arabidopsis* flowers.

**Results:**

In *as1* mutant flowers, the sepal abscission zones are displaced into inverted V-shaped positions, leaving behind triangular stubs of tissue when the organs abscise. Movement of the petal abscission zones is also apparent. Abscission of the medial sepals is delayed in *as1* flowers; loss of chlorophyll in the senescing sepals contrasts with proximal zones that remain green. AS1 has previously been shown to restrict expression of the *KNOX* gene, *BREVIPEDICELLUS* (*BP),* from the sepals. We show here that loss of BP activity in *as1* flowers is sufficient to restore the positions of the sepal and petal abscission zones, the sepal-receptacle boundary of the medial sepals and the timing of their abscission.

**Conclusions:**

Our results indicate that AS1 activity is critical for the proper placement of the floral organ abscission zones, and influences the timing of organ shedding.

## Background

The process of organ abscission allows plants to detach leaves, floral organs, fruit and seeds at specific points in their life cycles or in response to environmental cues. Genetic analysis in *Arabidopsis* has been particularly productive in revealing factors that activate the cell separation phase of floral organ abscission [1],[2]. Less is known about the regulatory circuits that control abscission zone differentiation at the boundaries between the outer floral organs and underlying receptacle [3],[4].

Several genes that regulate lateral organ and/or inter-organ boundaries also affect floral organ abscission. The transcriptional activators BLADE-ON-PETIOLE1 (BOP1) and BOP2 redundantly control patterning of the proximal regions of developing leaves and floral organs [5]–[7]. Anatomical evidence of abscission zone differentiation cannot be detected in *bop1 bop2* flowers and the sepals, petals and stamens remain strongly attached [8]. The BELL-type homeodomain transcription factor, ARABIDOPSIS THALIANA HOMEOBOX GENE1 (ATH1), represses growth in regions that will become the floral organ-receptacle boundaries and is required for stamen abscission zone formation [9]. HAWAIIAN SKIRT (HWS), an F-box protein, prevents the fusion of adjacent sepals and influences the timing of their abscission [10].

Five mutant alleles of *ASYMMETRIC LEAVES1* (*AS1*), a key determinant of polarity and cell fate in lateral organs [11], were identified through a screen for floral organ shedding mutants [12] (Additional file 1: Table S1). AS1, a MYB transcription factor, acts in conjunction with the AS2 LATERAL ORGAN BOUNDARIES domain (LBD) transcription factor to repress expression of a set of meristem-promoting *KNOTTED1-LIKE HOMEODOMAIN* (*KNOX*) genes during leaf development [13]–[15]. Ectopic activity of the BREVIPEDICELLUS (BP, also known as KNAT1), KNAT2 and KNAT6 homeodomain transcription factors has been genetically linked to the reduced size, shorter petioles and rumpled appearance of *as1* mutant leaves [13],[16]. The AS1-AS2 repressor complex recruits components of the Polycomb-repressive complex2 to the promoters of *BP* and *KNAT2*, to establish an inactive chromatin state in leaf cells [17]–[19].

AS1 also restricts expression of *BP* and *KNAT2* within developing flower primordia [13]. *BP* is expressed in the pedicel, receptacle, and organ boundaries of wild-type flowers, while *KNAT2* is primarily restricted to the floral organ boundaries and receptacle [13],[20]–[22]. In *as1* mutant flowers, which open prematurely due to their smaller sepals and petals, expression of *BP* and *KNAT2* expands into the sepals [13],[23]. Genetic analysis has shown that the combined loss of BP, KNAT2 and KNAT6 activity is sufficient to rescue the reduced organ size and open bud defects of *as1* mutant flowers [16].

BP directs growth of the receptacle, an expanded region of the pedicel [20]. *bp* flowers have slender, abbreviated pedicels due to radial constriction of the receptacle and reduced cell division [20],[23],[24]. Due to increased constriction on the abaxial side of the pedicel, *bp* pedicels bend down rather than pointing up [20]. BP also functions during floral organ abscission to prevent premature shedding and inhibit expansion of abscission zone cells [21],[25]. The abscission zones of *bp* flowers are notably enlarged, similar to those of flowers with constitutive expression of *INFLORESCENCE DEFICIENT IN ABSCISSION* (*IDA*) [26]. Signaling by the IDA peptide through the HAESA (HAE) and HAESA-LIKE2 receptor-like kinases has been proposed to activate organ abscission by inhibiting BP activity [25],[27],[28].

Here we show that AS1 controls the placement of the sepal and petal organ abscission zones in *Arabidopsis* flowers. The medial sepals of *as1* mutant flowers are most affected: delayed organ abscission occurs along the edges of a triangular-shaped proximal domain with altered identity. We further show that loss of BP activity is sufficient to rescue the abscission-related defects of *as1* mutant flowers.

## Methods

### Plants

Five *as1* (formerly known as *bibb*) mutants were previously identified through a genetic screen for organ shedding mutants [12] (Additional file 1: Table S1); all mutants are of the Landsberg *erecta* (L*er*) ecotype. The F1 progeny of a cross between the *as1-21* and *as1-20* mutants showed abscission defects, consistent with an allelic relationship between the mutations. *as1-1* (L*er*; CS146) and *bp-1* (L*er*; CS30) seeds were obtained from ABRC (Columbus, OH). Plants homozygous for *as1-20* were distinguished with a CAPS (cleaved amplified polymorphic sequence) [29] marker based on a BccI site present in the wild-type allele of *AS1*. The oligos used to amplify this region of the *AS1* gene are described in Additional file 2: Table S2. Plants were grown at 21°C with 50% humidity and a 16 hour photoperiod.

### Microscopy and marker analyses

Wild-type and mutant flowers were fixed as previously described [30], and prepared for scanning electron microscopy using a Tousimis Samdri-790 critical point dryer (Tousimis, Rockville, MD) and EMS 550 sputter coater equipped with a film thickness monitor (EMS, Hatfield, PA). Samples were examined using an accelerating voltage of 10 kV in a FEI XL-30 scanning electron microscope (FEI, Hillsboro, OR). Young flowers (stages 8–12) were dissected and reimaged to determine their developmental stage [31].

A transgenic *HAE::GUS* line [32] was crossed to the *as1-20* and *as1-20 bp-1* mutants to generate single and double mutants carrying this abscission zone marker. For β-glucuronidase assays, wild-type and mutant flowers were fixed and stained as described [33] with minor modifications. Digital images were taken with a Stemi SV11 dissecting microscope and Axiocam HR camera (Carl Zeiss, Germany) or PowerShot SX160 IS (Canon, Melville, NY). Image brightness and contrast were adjusted with Photoshop CS6 (Adobe, Mountain View, CA). Abscission zone displacement was measured using NIH ImageJ [34].

### Molecular biology

Genomic DNA samples were prepared from wildtype (Ler) and two independent *as1-21* mutant plants using the DNeasy Plant Mini Kit (Qiagen, Venlo, Netherlands). Regions of the *AS1* gene were PCR amplified as described in Additional file 2: Table S2. PCR products were purified using the Zymoclean Gel DNA recovery kit (Irvine, CA) and sequenced (MacrogenUSA, Rockville, MD). Sequences were analyzed using Geneious R6.1 software (Biomatters, Auckland, NZ).

## Results

### Identification of a new mutation in the *AS1* MYB domain

**Figure 1:**
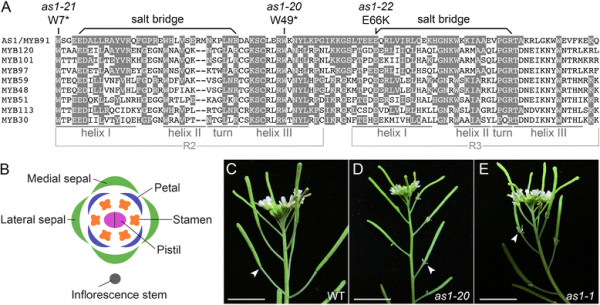
**Mutations in*AS1*delay sepal abscission. A**: Sequence alignment of the DNA-binding domain of AS1 and related *Arabidopsis* R2R3 MYB proteins. Conserved amino acid residues are shaded. The pairs of acidic glutamic acid (E) and basic arginine (R) residues that form salt bridges in the R2 and R3 repeats are linked by bars. The positions of three *as1* mutations that alter organ abscission are indicated. The *as1-20* (reference allele for this study) and *as1-21* mutations introduce stop codons in place of codons specifying tryptophan. The *as1-22* mutation replaces a conserved glutamic acid with a lysine; the affected residue is an integral part of the salt bridge formed in the R3 repeat of AS1. **B**: Diagram of a wild-type flower illustrating the orientation of the medial and lateral sepals with respect to the inflorescence stem. **C**: Wild-type inflorescence. Flowers shed their outer organs (stage 16), leaving the developing fruit behind. Scale bar, 1 cm. **D/E**: *as1-20***(D)** and *as1-1***(E)** mutant inflorescences. Mutant flowers typically retain their medial sepals until after the fruit is fully elongated (stage 17). Scale bars, 1 cm.

AS1 is one of 126 proteins in *Arabidopsis* with a MYB domain composed of R2 and R3 repeats [35],[36]. Each repeat consists of three helices, of which the second and third form a helix-turn-helix motif that binds DNA (Figure 1A). To characterize the role of *AS1* in organ abscission, we selected *as1-20* (formerly known as *bibb-1*) [12] and *as1-1*[11],[23],[37] as reference alleles (Additional file 1: Table S1). The associated nonsense mutation for *as1-20* is in the third helix of the AS1 R2 repeat (Figure 1A) [12]. The *as1-21* mutant (formerly known as *bibb-2*), was found to introduce a nonsense mutation in the first helix of the AS1 R2 repeat (Figure 1A).

### The *as1-22* mutation disrupts a salt bridge in the DNA binding domain

Within each repeat of eukaryotic R2R3 MYB proteins, an acidic amino acid residue in the first helix forms a salt bridge with a basic amino acid residue in the turn adjacent to the third helix [38] (Figure 1A). The glutamic acid and arginine residues that form salt bridges in R2R3 MYB proteins are conserved in more than 97% of the *Arabidopsis* family members surveyed [35] (Figure 1A). Of the *as1* alleles that introduce point mutations [12],[37], *as1-22* (formerly known as *bibb-5*) [12] represents the only missense mutation identified to date. Replacement of the affected glutamic acid with lysine, a basic residue, would prevent salt bridge formation in the R3 repeat of the as1-22 mutant protein (Figure 1A). An alanine substitution of the corresponding glutamic acid within the R3 repeat of the c-Myb transcription factor abolishes both DNA binding and transcriptional activation [38].

### Mutations in *AS1* delay shedding of the medial sepals

*Arabidopsis* flowers contain pairs of medial and lateral sepals, four petals and six stamens, which are shed shortly after fertilization (stage 16) (Figure 1B, C) [31]. In each of the *as1* mutants known to alter organ abscission (Figure 1D, E; Additional file 1: Table S1; Additional file 3: Figure S1), shedding of the medial sepals is delayed until after the fruit is fully elongated (early stage 17). When *as1* flowers are touched during the normal period of abscission (stage 16), the medial sepals remain firmly attached. Of 30 *as1-1* flowers (early to mid stage 17) surveyed after touching, 80% (48/60) of the medial sepals remained attached compared to 0% (0/20) for 10 wild-type flowers. Abscission of the lateral sepals is also delayed in some *as1-21*, *as1-22* and *as1-23* flowers (Additional file 3: Figure S1B, E).

### The sepal and petal abscission zones of *as1* flowers are displaced

**Figure 2:**
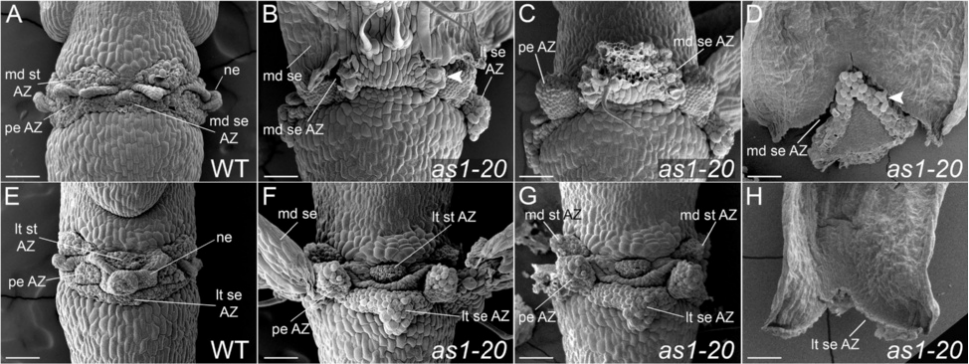
**The positions of the sepal and petal abscission zones are altered in*as1*mutant flowers.** Scanning electron micrographs of wild-type and mutant flowers (stage 17). Medial **(A-D)** and lateral **(E-H)** views are shown. **A/E**: After the outer organs are shed in wild-type flowers, the sepal (se) abscission zone (AZ) appears as a continuous ring adjacent to the pedicel. Individual petal (pe) and stamen (st) AZ regions can be distinguished. The remaining AZ cells have a rounded appearance. Nectary (ne) tissue is sandwiched between the stamen and petal AZs. **B**: Abscission of the medial (md) sepals is delayed in *as1* flowers. Cell separation first occurs at the proximal edges of an inverted V-shaped region. Cell expansion is apparent in the sepal AZ cells that remain associated with this region (see arrowhead). **C**: Flower shown in **(B)** with medial sepal removed. Stubs of tissue are visible at the petal attachment sites. **D**: Inner view of an *as1* medial sepal. Expansion of cells within an inverted V-shaped AZ is apparent (see arrowhead). **F**: Rounded cells are present at the tips of the stubs found at the petal attachment sites. Triangular-shaped regions of tissue are present at the lateral (lt) sepal attachment sites. **G**: Flower shown in **(F)** with the medial sepals removed. Positioning of the lateral stamen AZs in *as1* flowers is not detectably altered; the medial stamen AZs appear to be slightly displaced. **H**: Inner view of an *as1* lateral sepal (stage 16). An inverted V-shaped AZ is present. Scale bars, 100 μm.

**Figure 3:**
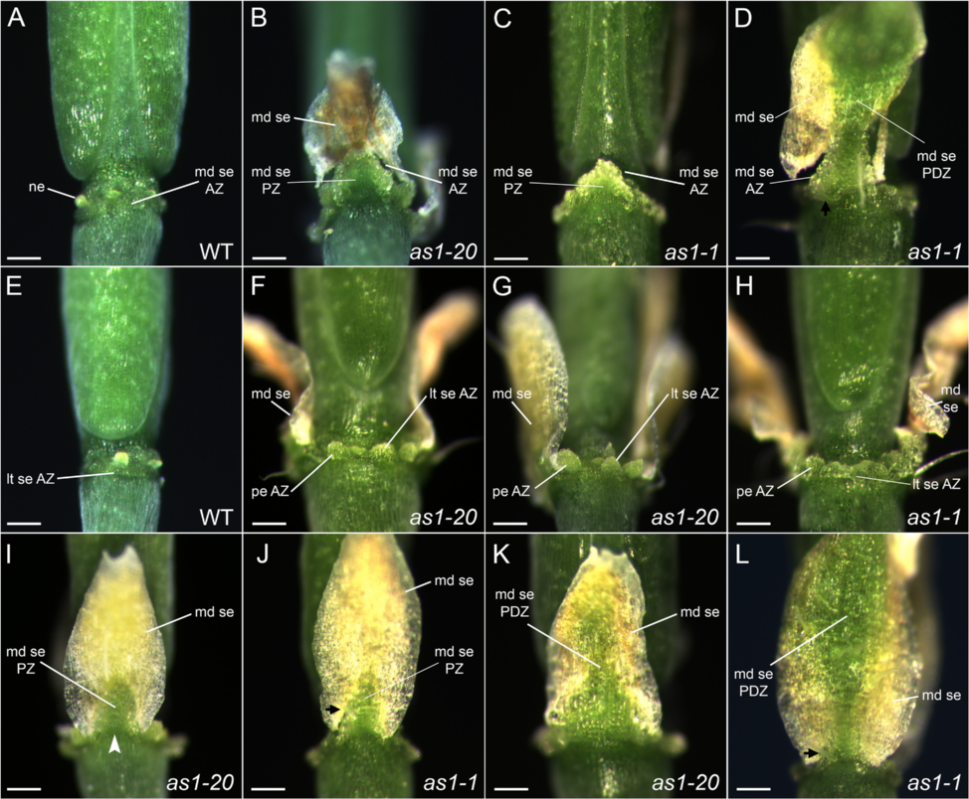
**Mutations in*AS1*alter the proximal domains of the medial sepals.** Medial **(A-D, I-L)** and lateral **(E-H)** views of wild-type and *as1* mutant flowers (stage 17). **A/E**: Wild-type flowers. **B-D**: Detachment of the medial sepals of *as1-20***(B)** and *as1-1***(C)** flowers usually occurs at inverted V-shaped abscission zones (AZ). The triangular-shaped regions of proximal zone (PZ) tissue that remain behind do not senesce. In some *as1* flowers **(D)**, the sides of the sepals abscise along the edges of larger proximal-distal zones (PDZ) that remain green. **F-H**: Shedding of the lateral sepals and petals of *as1-20***(F/G)** and *as1-1***(H)** flowers leaves behind tissue stubs that remain green. **I/J**: The altered PZs of *as1-20***(I)** and *as1-1***(J)** medial sepals can be visualized during the extended senescence that occurs prior to organ abscission. The constriction of growth associated with the sepal-receptacle boundary in wild-type flowers is located at the base of the altered PZs (see arrowhead, **I**). **K/L**: Substantially larger, hourglass-shaped PDZs are observed in some *as1-20***(K)** and *as1-1***(L)** medial sepals. Scale bars, 200 μm.

After abscission (stage 17), the positions of the floral organ abscission zones can be easily visualized in wild-type flowers (Figure 2A, E; Figure 3A, E). Discrete domains of the abscission zone cells that remain with the plant body are present for the petals and stamens, along with a band of sepal abscission zone cells that encircles the receptacle. Placement of the sepal abscission zones is altered in *as1* flowers (stage 17). Instead of developing at the sepal base, abscission zones are usually formed in an inverted V-shape within the proximal regions of *as1* medial and lateral sepals (Figure 2B-D, F-H; Figure 3B; Additional file 3: Figure S1). When the sepals are shed, triangular regions of tissue remain attached at the bases of *as1* flowers (Figure 2D, F, H; Figure 3C, F, G; Additional file 3: Figure S1F). Displacement of the medial sepal abscission zones (Figure 2D; Figure 3B-D) is more pronounced relative to that of the lateral sepal abscission zones (Figure 2F; Figure 3F-H). The distalmost position of *as1-20* medial abscission zones is 279 ± 15 μm (n = 5) from the sepal base, compared to 133 ± 13 μm (n = 4) for *as1-20* lateral abscission zones. In *as1-1* flowers, the medial sepal abscission zones were displaced 268 ± 34 μm (n = 7) compared to 107 ± 11 μm (n = 6) for the lateral abscission zones.

The positions of the petal abscission zones are also shifted in *as1* flowers (Figure 2C, F; Figure 3F-H; Additional file 3: Figure S1). After abscission, the tissue stubs that remain at the petal attachment sites are 126 ± 18 μm (n = 8) for *as1-20* flowers and 92 ± 8 μm (n = 8) for *as1-1* flowers. While placement of the lateral stamen abscission zones appears to be unaffected in *as1* flowers (Figure 2F), small tissue projections are sometimes observed at the attachment sites for the medial stamens (Figure 2G).

### The proximal regions of *as1* medial sepals show an altered identity

The outer organs of wild-type flowers begin to wither and senesce shortly before abscission (stage 16), but are still turgid when they detach [39]. In *as1* flowers (stage 17), due to the delay in abscission of the medial sepals, two distinctive patterns of chlorophyll loss are observed after the extended senescence period (Figure 3B, D, I-L). The primary pattern is that chlorophyll is retained in a triangular-shaped proximal zone and lost elsewhere in *as1* medial sepals (Figure 3I, J). Larger proximal-distal green zones are also observed in some *as1* medial sepals (Figure 3K, L). Of 31 *as1-1* medial sepals surveyed, 19 (61%) had green proximal zones with a triangular shape (Figure 3I, J). The other 12 (39%) had green proximal-distal zones with an hourglass shape (Figure 3K, L). If the neck of the altered proximal-distal zone is narrow, only the triangular-shaped proximal region remains attached after abscission (data not shown). If it is wide, the sides of the medial sepal begin to abscise, starting from the proximal edges (Figure 3D).

### Establishment of the medial sepal-receptacle boundary is altered in *as1* flowers

**Figure 4:**
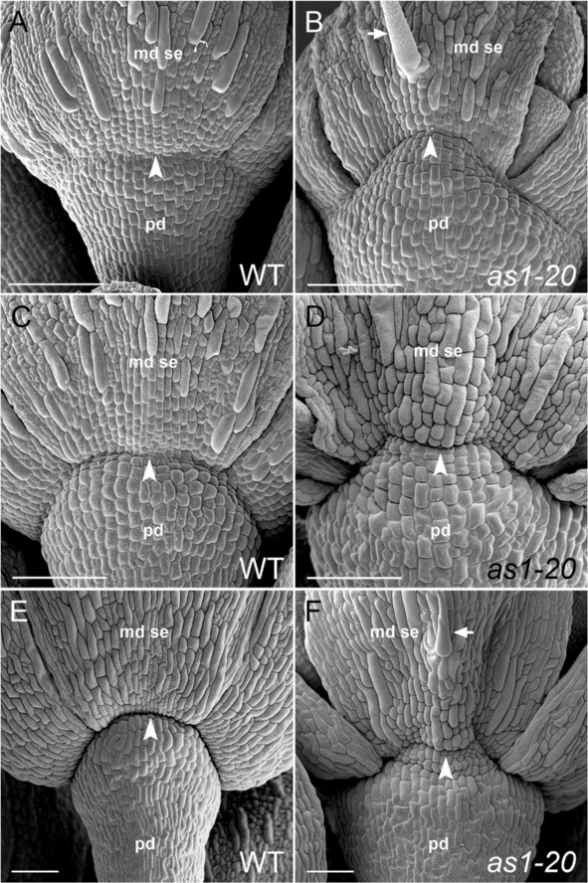
**Mutations in*AS1*affect the medial sepal-receptacle boundary.** Scanning electron micrographs of wild-type and mutant sepal primordia. Boundary regions between the medial sepal (md se) primordia and underlying flower pedicels (pd) are indicated by arrowheads. **A**: In wild-type flowers (stage 8), a crease marks the site of the future sepal-receptacle (distal pedicel) boundary. **B**: This incipient boundary is shifted in *as1* medial sepal primordia (stage 8). **C**: As wild-type flowers (stage 9) develop, the surface of the sepal is uniformly rounded. **D**: The central, proximal region of *as1* sepal primordia (stage 9) protrudes from the surface. **E**: A well-defined boundary between the sepals and receptacle is present in wild-type flowers (stage 12). Trichomes are not observed in the proximal regions of sepals. **F**: The boundary between the medial sepals and pedicel is altered in *as1* flowers (stage 12). Trichomes (see arrow, also in **(C)**) are frequently observed in the central, proximal regions of *as1* medial sepals. Scale bars, 100 μm.

In wild-type flowers, a crease marking the future sepal-receptacle boundary is apparent at the bases of the sepal primordia by stage 8 (Figure 4A; stages assigned according to [31]). As development continues (stage 9), constriction of growth in this region compared to the cells found in the sepal primordia above and floral receptacle below (Figure 4C) results in the formation of a clearly defined boundary (Figure 4E, stage 12) [20],[40]. In *as1* mutant flowers (stage 8), the position of the initial crease is distally displaced at the midpoints of the medial sepal primordia (Figure 4B). Although some growth suppression is evident (Figure 4D, stage 9), the medial sepal-receptacle boundary regions of *as1* flowers (Figure 4F, stage 12) are disorganized and less pronounced than those of wild-type (Figure 4E). As *as1* flowers mature (stage 15), the slight displacement of this boundary in the medial sepals can still be detected (Additional file 4: Figure S2B) compared to wild-type (Additional file 4: Figure S2A). The lateral sepal-receptacle boundary regions of *as1* flowers (stage 15) resemble those of wild-type flowers (Additional file 4: Figure S2E, F).

Whereas wild-type sepal primordia are rounded (Figure 4C, E), the central-proximal domains of *as1* medial sepal primordia become ridged (Figure 4D, F). Trichomes are frequently present in these regions of *as1* sepals (Figure 4B, F; Figure 3J, L; Figure 2B), and are not observed in the corresponding areas of wild-type sepals (Figure 4A, C, E). The altered appearance of the proximal domains of *as1* medial sepals corresponds with the regions that retain chlorophyll as the rest of the sepal senesces (Figure 3I-J). Abscission occurs along the distal edges of these proximal zones (Figure 3B; Figure 2D) rather than coinciding with the regions where growth is constricted at their bases (Figure 2B; Figure 3I).

### The abscission zone defects of *as1* flowers are BP-dependent

**Figure 5:**
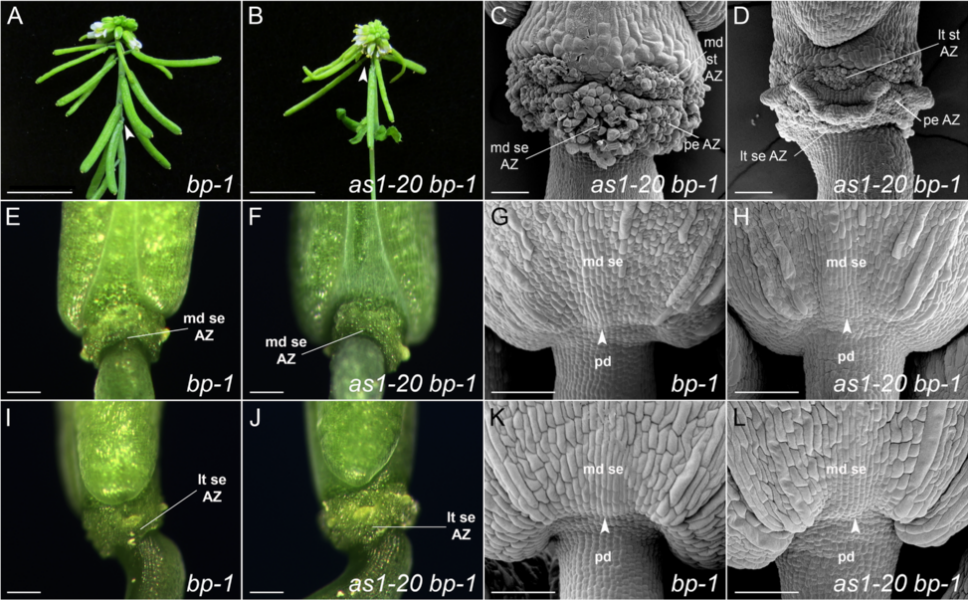
**Loss of*BP*restores abscission zone placement and the position of the medial sepal-receptacle boundary in*as1*mutant flowers. A**: *bp* inflorescence. The flower pedicels are short, slender and point downward. **B**: *as1 bp* inflorescence. Abscission of the medial sepals is not delayed. **C/D**: Scanning electron micrographs of *as1 bp* flowers (stage 17) showing medial **(C)** and lateral **(D)** views. Placement of the abscission zones in *as1 bp* flowers **(D)** is similar to that of wild-type (see Figure 2A, E). The abscission zones of *as1 bp* flowers **(C)**, as previously reported for *bp-101, bp-3 and bp-10* flowers [21],[25], show increased cell expansion during fruit development. **E/F/I/J**: Medial **(E/F)** and lateral **(I/J)** views of *bp* and *as1 bp* flowers (stage 17). The positions of the enlarged abscission zones of *bp***(E/I)** and *as1 bp***(F/J)** flowers are similar to those of wild-type flowers (see Figure 3A, E). **G/H/K/L**: Scanning electron micrographs of *bp* and *as1 bp* medial sepal primordia. Boundary regions between the medial sepal primordia and underlying flower pedicels are indicated by arrowheads. **G:** In *bp* flowers (stage 9), the pedicels are slender and the receptacle region beneath the floral organs fails to expand. **H:** Displacement of the sepal-receptacle boundary is not evident in *as1 bp* flowers (stage 9) or *bp* flowers **(G)**. **K/L**: Due to radial constriction of the pedicel, the boundary between the sepals and receptacle is less distinct in *bp***(K)** and *as1 bp***(L)** flowers (stage 11). Scale bars: 1 cm **(A/B)**; 100 μm **(C/D/G/H/K/L)**; 200 μm **(E/F/I/J)**.

To determine whether BP activity contributes to the displaced abscission zones of *as1* flowers, we generated the *as1-20 bp-1* double mutant. *bp-1* is a null allele due to a deletion that spans the entire locus [20],[24]. As previously reported for the *as1-1 bp-2* double mutant [23], the overall appearance of the *as1-20 bp-1* inflorescences is largely additive with respect to those of the single mutants. Like the *bp-1* mutant (Figure 5A, E, I) [24], *as1-20 bp-1* mutant inflorescences have abbreviated internodes and slender, short, downward-pointing pedicels (Figure 5B, F, J). As in the *as1-20* mutant (Figure 1D), the reduced size of the sepals and petals of *as1-20 bp-1* flowers results in premature bud opening and exposure of the developing stamens and carpels (Figure 5B).

Loss of BP activity is, however, sufficient to restore the positions of the sepal and petal abscission zones in *as1* flowers (Figure 5C, D, F, J) as well as the timing of medial sepal abscission (Figure 5B). As in wild-type flowers, distinct abscission zones are initially observed in *as1 bp* flowers after organ shedding (Figure 5D; Figure 2A, E). As fruit maturation progresses (stage 17), the sepal abscission zones of *as1 bp* flowers become enlarged and have a disorganized appearance (Figure 5C). This result is consistent with the abscission zone enlargement that is characteristic of *bp* flowers [21],[25].

In younger *as1 bp* and *bp* flowers (stage 9), the positions of the sepal-receptacle boundaries are not distally displaced (Figure 5G, H) as they are in *as1* flowers (Figure 2B, D). In addition, the central-proximal regions of *as1 bp* medial sepals are not ridged, nor do they contain ectopic trichomes (Figure 5H, L). These results suggest that the altered identity of the proximal zone and the shifted sepal-pedicel boundary in *as1* flowers are also BP-dependent.

Due to radial constriction of the receptacle, the creases at the bases of *bp* and *as1 bp* sepal primordia (stage 11) are notably less distinct (Figure 5K, L) than those of wild-type flowers (Figure 2E). By promoting expansion of the receptacle in wild-type flowers, BP activity influences the definition of the sepal-pedicel boundary.

### Expression of the *HAE::GUS* abscission zone marker is altered in *as1* flowers

**Figure 6:**
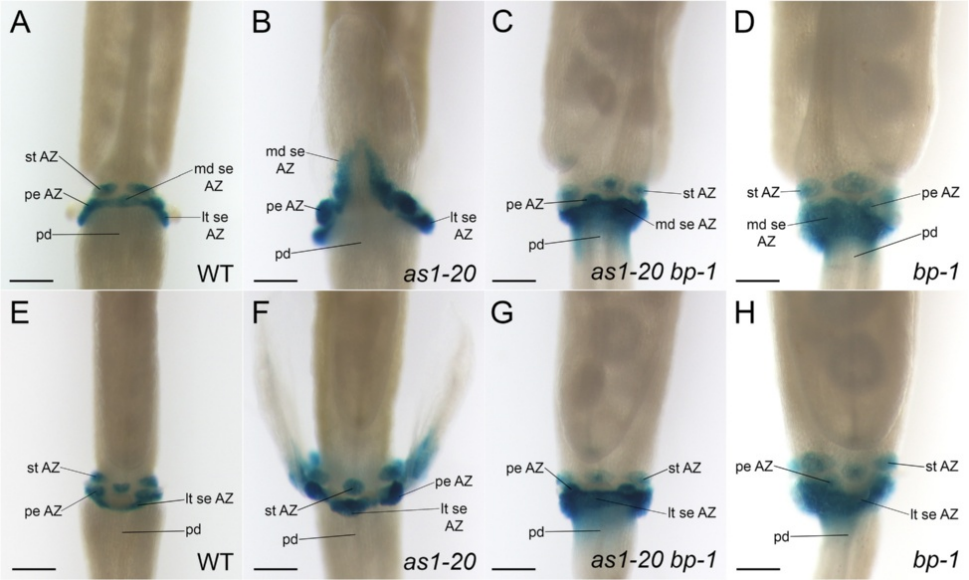
**Expression of the*HAE::GUS*abscission zone marker is displaced in*as1*medial sepals.** Images of wild-type and mutant flowers (stage 17) histochemically stained for β-Glucuronidase (GUS) activity. Medial **(A-D)** and lateral **(E-H)** views are shown. **A/E**: In wild-type flowers, *HAE* regulatory regions direct expression of *GUS* in discrete domains of the remaining sepal (se), petal (pe) and stamen (st) abscission zone cells. **B/F**: In *as1-20* flowers, *GUS* is expressed in an inverted V-shaped proximal region of the medial sepals **(B)**, and in the remaining petal and lateral sepal stubs **(B/F)**. **C/D/G/H**: Expression of *GUS* expands beyond the enlarged sepal abscission zones of *as1-20 bp-1***(C/G)** and *bp-1***(D/H)** flowers into the distal pedicels. Scale bars: 200 μm.

We have presented morphological evidence that the placement of sepal and petal abscission zones is shifted in *as1* mutant flowers (Figures 2 and 3; Additional file 3: Figure S1). To examine the profile of a known marker of floral organ abscission zones in *as1* flowers, we crossed the *as1-20* mutant to a transgenic plant carrying a fusion of the *HAE* promoter to the *β-Glucuronidase* (*GUS*) reporter gene [32],[41]. In wild-type *HAE::GUS* flowers (stage 17), expression of *GUS* persists in the remaining floral organ abscission zone cells in discrete domains that mark the sepal, petal and stamen attachment sites (Figure 6A, E). In *as1-20* flowers (stage 17), *HAE* regulatory regions usually direct *GUS* expression in an inverted V-shape in the proximal zone of the medial sepals (Figure 6B). Like the delayed progression of cell separation (Figure 2B, D; Figure 3B, D), expression of this marker originates at the proximal edges of *as1-20* medial sepals (Additional file 5: Figure S3A, B) and usually expands distally until the lines intersect at the sepal midpoint (Additional file 5: Figure S3C-F; Figure 6B). *GUS* expression is also found in the tissue remnants that remain at the petal and lateral sepal attachment sites of *as1-20* flowers (Figure 6B, F).

We have shown that loss of BP activity is sufficient to restore the positions of the sepal and petal abscission zones in *as1-20* flowers. To determine whether the expression profile of the *HAE::GUS* abscission zone marker is also rescued in *as1-20 bp-1* flowers, mutants carrying this marker were generated. We discovered that, as in wild-type flowers (Figure 6A, E), *GUS* is expressed in the remaining sepal and petal abscission zone cells at the bases of *as1-20 bp-1* (Figure 6C, G) as well as *bp-1* (Figure 6D, H) flowers (stage 17). Interestingly, the band of *GUS* expression corresponding to the sepal abscission zone cells is conspicuously wider and diffuses into the distal pedicel of both *as1-20 bp-1* (Figure 6C, G) and *bp-1* flowers (Figure 6D, H), in correspondence with the enlarged abscission zones of these mutant flowers.

Taken together, these results provide molecular evidence that sepal and petal abscission zones are distally displaced in *as1* flowers, and that this displacement is BP-dependent.

## Discussion

We report here a novel role for the AS1 MYB transcription factor in establishing the positions of the sepal and petal abscission zones. Three aspects of the altered pattern of sepal abscission in *as1* flowers are noteworthy. First, maximal abscission zone displacement occurs at the midpoint of each *as1* sepal (Figures 2 and 3). Second, the abscission zone midpoints of the medial sepals are shifted distally about twice as far as those of the lateral sepals (Figure 2B, F). Third, expression of the *HAE::GUS* abscission zone marker is not only displaced but shows a late onset in *as1* medial sepals (Additional file 5: Figure S3), which is mirrored by a significant delay in abscission (Figure 1D, E). These results suggest that the loss of AS1 activity primarily affects the position of abscission zone cell specification, and that the degree of displacement is influenced by orientation with respect to the inter-sepal boundary regions as well as the medial-lateral axis of the flower (Figure 1B). The delay in detachment of *as1* medial sepals may represent an alteration in either the timing of abscission zone specification or of the subsequent activation of cell separation.

Our analysis of *as1* flowers reveals that proximal zones within the medial sepals have an altered identity. As the attached organs senesce (stage 17), a distinct demarcation appears between proximal regions that remain green and the rest of the organ that loses its color as chlorophyll is catabolized (Figure 3I-L). Trichomes are frequently observed in the central, ridged proximal zones of *as1* medial sepals (Figure 2B; Figure 4B, F) and are not found within the corresponding rounded areas of wild-type sepals (Figure 4 A, C, E). The displaced abscission zones of *as1* medial sepals outline the distal edges of these altered proximal zones (Figure 3B). The region of constricted growth that defines the medial sepal-receptacle boundary of wild-type flowers [40],[42] is disorganized and slightly shifted at the bases of *as1* medial sepals (Figure 4; Additional file 4: Figure S2; Figure 2B). It is intriguing that the growth suppression associated with this boundary can be uncoupled from abscission zone differentiation in *as1* flowers. These results suggest that the sepal-receptacle boundary is disrupted in *as1* flowers but not completely dismantled.

Displacement of the sepal and petal abscission zones in *as1* flowers is BP-dependent (Figures 5 and 6). In wild-type flowers, the AS1-AS2 repressor complex restricts the *BP* expression domain to the pedicel, receptacle and organ boundary regions of the floral organ primordia [13]. Although the combined loss of BP, KNAT2 and KNAT6 activity is required to restore normal sepal and petal size in *as1* flowers [16], loss of BP alone is sufficient to rescue the shifted position of the sepal-receptacle boundary and the altered features of the proximal zone associated with the displaced abscission zones of *as1* medial sepals (Figure 5).

An interesting parallel to our results is the AS1-mediated regulation of *BP* expression during fruit patterning. *Arabidopsis* fruit are composed of two carpel valves attached to a medial replum [43]. Seed dispersal is facilitated by dehiscence zones that form at the valve-replum boundaries [30]. Within the developing gynoecium, AS1 restricts expression of a *BP::GUS* marker to the region that will become the replum [44]. Compared to wild-type fruit, *as1* mutant fruit contain a wider replum and narrower valves, and show expansion of the replum domain of *BP* expression as well as ectopic expression of *BP* in the valves [44]. Loss of BP activity was found to partially restore the respective sizes of the repla and valves in *as1 bp* fruit [44]. Through its regulation of the valve-replum boundary, AS1 controls the position of the fruit dehiscence zones. In *as1* fruit, the dehiscence zones are shifted laterally due to expansion of the replum [44]. We propose that AS1 similarly regulates placement of the floral organ-receptacle boundaries and thereby the positions of the sepal and petal organ abscission zones.

BP plays dual roles in regulating organ abscission. In addition to influencing the specification sites of abscission zone cells, BP prevents premature abscission by inhibiting the cell separation phase of abscission [25]. Signaling through the IDA-HAE/HSL2 pathway has been proposed to relieve the BP-mediated repression of organ abscission [25]. *bp* and *as1 bp* flowers develop enlarged, disorganized sepal abscission zones (Figure 5C, E) that resemble those of flowers constitutively expressing IDA [21],[25],[26]. We have found that expression of a *HAE::GUS* marker expands into the receptacles of *bp* and *as1 bp* flowers (Figure 6C, D, G, H). These results suggest that BP may control sepal abscission zone size by restricting *HAE* expression from the receptacle.

Our study provides fresh evidence to support the links between organ boundary formation and abscission zone development. Unlike ATH1 and BOP1/BOP2, which are required for some or all of the floral organ abscission zones to form [8],[9], AS1 and BP appear to play a more indirect role in abscission zone specification by regulating their positions. Our observation that displacement of the sepal abscission zones in *as1* flowers is less affected at the sepal margins than at the sepal midpoint (Figure 2D, H) suggests that factors active at the inter-sepal boundaries also influence abscission zone differentiation. The growth suppression that occurs at inter-organ boundaries has been previously found to affect the establishment of lateral organ boundaries. Flowers with mutations in the *CUP-SHAPED COTYLEDON1* (*CUC1*) and *CUC2* genes have fused sepals, but their sepal-receptacle boundaries are not altered [45]. However, reduction of CUC activity further obscures the definition of the sepal-receptacle boundaries of *ath1* flowers [9]. Further dissection of the complex regulatory networks that establish lateral and inter-organ boundaries is expected to uncover critical connections between the definition and positioning of the receptacle boundary, proximal-distal organ patterning, and floral organ abscission zone development.

## Conclusions

We have identified a novel role for AS1 in establishing the positions of the sepal and petal abscission zones. In *as1* mutant flowers, the sepal and petal abscission zones are displaced distally, detachment of the medial sepals is significantly delayed, and proximal domains within the medial sepals show an altered identity. Loss of BP activity rescues the abscission zone and proximal domain defects of *as1* flowers. Our results suggest that further advances in understanding the process of floral organ abscission zone development can be made by analyzing the network of genes known to control organ boundaries and proximal-distal patterning in *Arabidopsis* leaves.

## Competing interests

The authors declare that they have no competing interests.

## Authors’ contributions

SL conceived and coordinated this study. CG, MC, WG and SL performed SEMs. CG photographed plants and carried out marker analyses. DW and WG sequenced the *as1-21* allele. CG, MC, WG and DW prepared figures; SL wrote the manuscript. All authors read and approved the final manuscript.

## Additional files

## Supplementary Material

Additional file 1: Table S1. Alleles of *ASYMMETRIC LEAVES1* (*AS1*) that alter organ abscission [12],[37]. Click here for file

Additional file 2: Table S2. *AS1* oligos used in this study. Click here for file

Additional file 3: Figure S1. Shedding of the lateral sepals is delayed in some *as1* flowers. Medial **(A/B/E-H)** and lateral **(C/D)** views of mutant flowers (stage 17). Shedding of the medial sepals is delayed in *as1-21***(A/B)**, *as1-101***(E)**, *as1-23***(F)**, and *as1-22***(G/H)** flowers. Shedding of the lateral sepals is delayed in some *as1-21***(B/D)**, *as1-23***(F)**, and *as1-22* flowers. Of 10 flowers (early to mid stage 17) surveyed per genotype after light touching, all of the medial sepals (20 of 20) remained attached in *as1-21* and *as1-23* flowers, while 75% (15 of 20) and 70% (14 of 20) remained in *as1-22* and *as1-101* flowers, respectively. None of the lateral sepals remained attached in *as1-101* (0 of 20) or *as1-1* (0 of 60) flowers; 20% (4 of 20), 15% (3 of 20), and 15% (3 of 20) remained in *as1-21*, *as1-22*, and *as1-23* flowers, respectively. Scale bars, 200 μm. Click here for file

Additional file 4: Figure S2. Displacement of the receptacle boundary is not detected in the lateral sepals of *as1-20* flowers. Medial **(A, B)** and lateral **(C, D)** views of wild-type and *as1-20* flowers (stage 15). In comparison with wild-type flowers **(A/C)**, placement of the sepal-pedicel boundary is affected in the medial (**B**, see arrow) but not the lateral **(D)** sepals of *as1-20* flowers. Scale bars, 200 μm. Click here for file

Additional file 5: Figure S3. Expression of the *HAE::GUS* marker progresses distally from the proximal edges of *as1* medial sepals. Medial views of *as1-20* mutant flowers (stage 17) histochemically stained for β-Glucuronidase (GUS) activity. Expression of *GUS* initiates at the proximal margins of the medial sepals **(A, B)**. Stripes of *GUS* expression extend from each origin in a distal direction **(B, C)** until they intersect **(D)** to outline the edges of an inverted V-shaped proximal domain. In some *as1-20* medial sepals **(E, F)**, stripes of GUS expression expand distally toward the sepal tip without intersecting in a proximal region. Click here for file
