# Supplementary material for: ASYMMETRIC LEAVES1 regulates abscission zone placement in Arabidopsis flowers
Source: BMC Plant Biol. 2014 Jul 20;14:195. doi: 10.1186/s12870-014-0195-5 (PMC4223632; doi:10.1186/s12870-014-0195-5)
Supplement: Additional file 1: Table S1. — Alleles of ASYMMETRIC LEAVES1 (AS1) that alter organ abscission [12],[37]. [file s12870-014-0195-5-S1.pdf]

Table S1. Alleles of *ASYMMETRIC LEAVES1* (*AS1*) that alter organ abscission

	Mutation	Sequence references
as1-20	W49*	*bib-1* [12]
as1-21	W7*	*bib-2* [this study]
as1-22	E66K	*bib-5* [12]
as1-23	Q171*	*bib-6* [12]
as1-101	Q107*	[37]; *bib-3*=*bib-4* [12]
as1-1	Deletion in codon 231 results in frameshift and L235*	[37]
