# Supplementary material for: ASYMMETRIC LEAVES1 regulates abscission zone placement in Arabidopsis flowers
Source: BMC Plant Biol. 2014 Jul 20;14:195. doi: 10.1186/s12870-014-0195-5 (PMC4223632; doi:10.1186/s12870-014-0195-5)
Supplement: Additional file 2: Table S2. — AS1 oligos used in this study. [file s12870-014-0195-5-S2.pdf]

Table S2. *AS1* oligos used in this study.

Purpose	Sequence
as1-20 genotyping	5'-GAGAGACAACGTTGGAGTGGTGAAG-3'
"	5'-CAGCAGCAGGGACAACGTTAGAC-3'
Sequence analysis: region 1	5'-ATGAAAGAGAGACAACGTTGGAGTG-3'
"	5'-GACGGTTCAGGGGCGGTCTAATC-3'
Sequence analysis: region 2	5'-TCTGCTCTTCCTCTGTCAAAGACC-3'
"	5'-GGTTTTAAAAGCCTCTCTCACTTTG-3'
Sequence analysis: region 3	5'-AGACCATAAGAAAGAGGCTGCATG-3'
"	5'-TGAAGTGATAAATGGAACACAAAGG-3'
