# Supplementary figures and images for: ASYMMETRIC LEAVES1 regulates abscission zone placement in Arabidopsis flowers
Source: BMC Plant Biol. 2014 Jul 20;14:195. doi: 10.1186/s12870-014-0195-5 (PMC4223632; doi:10.1186/s12870-014-0195-5)

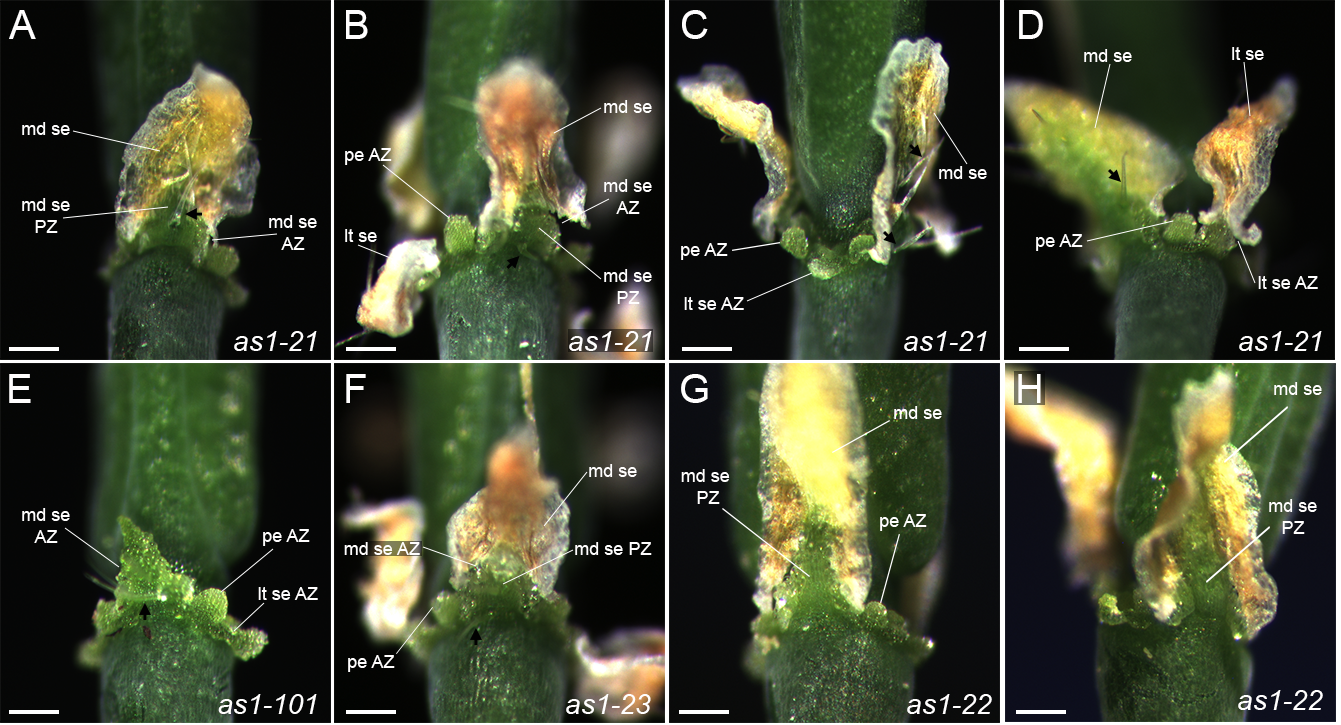

Supplement: Additional file 3: Figure S1. — Shedding of the lateral sepals is delayed in some as1 flowers. Medial (A/B/E-H) and lateral (C/D) views of mutant flowers (stage 17). Shedding of the medial sepals is delayed in as1-21(A/B), as1-101(E), as1-23(F), and as1-22(G/H) flowers. Shedding of the lateral sepals is delayed in some as1-21(B/D), as1-23(F), and as1-22 flowers. Of 10 flowers (early to mid stage 17) surveyed per genotype after light touching, all of the medial sepals (20 of 20) remained attached in as1-21 and as1-23 flowers, while 75% (15 of 20) and 70% (14 of 20) remained in as1-22 and as1-101 flowers, respectively. None of the lateral sepals remained attached in as1-101 (0 of 20) or as1-1 (0 of 60) flowers; 20% (4 of 20), 15% (3 of 20), and 15% (3 of 20) remained in as1-21, as1-22, and as1-23 flowers, respectively. Scale bars, 200 μm. [file s12870-014-0195-5-S3.tiff]

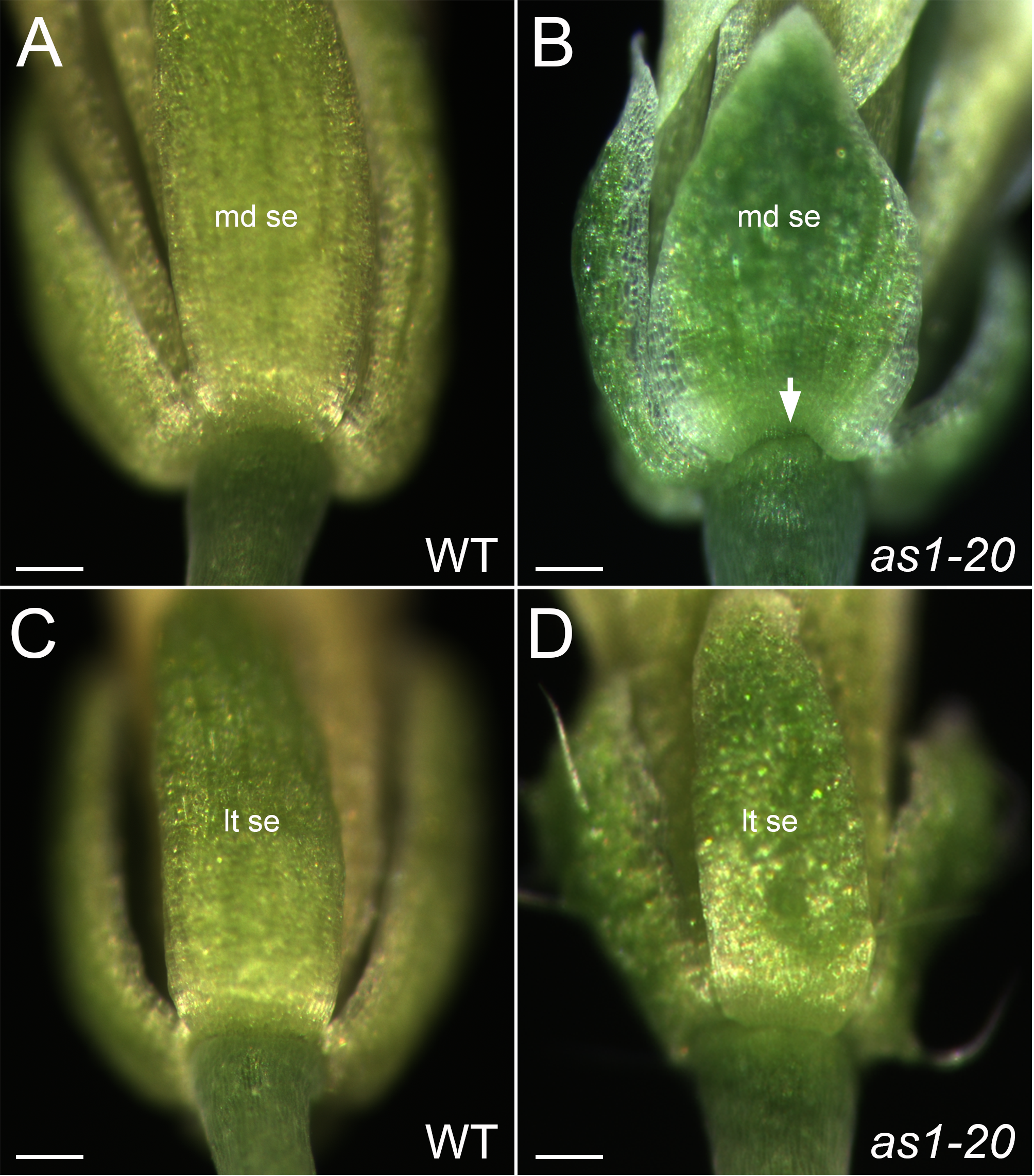

Supplement: Additional file 4: Figure S2. — Displacement of the receptacle boundary is not detected in the lateral sepals of as1-20 flowers. Medial (A, B) and lateral (C, D) views of wild-type and as1-20 flowers (stage 15). In comparison with wild-type flowers (A/C), placement of the sepal-pedicel boundary is affected in the medial (B, see arrow) but not the lateral (D) sepals of as1-20 flowers. Scale bars, 200 μm. [file s12870-014-0195-5-S4.tiff]

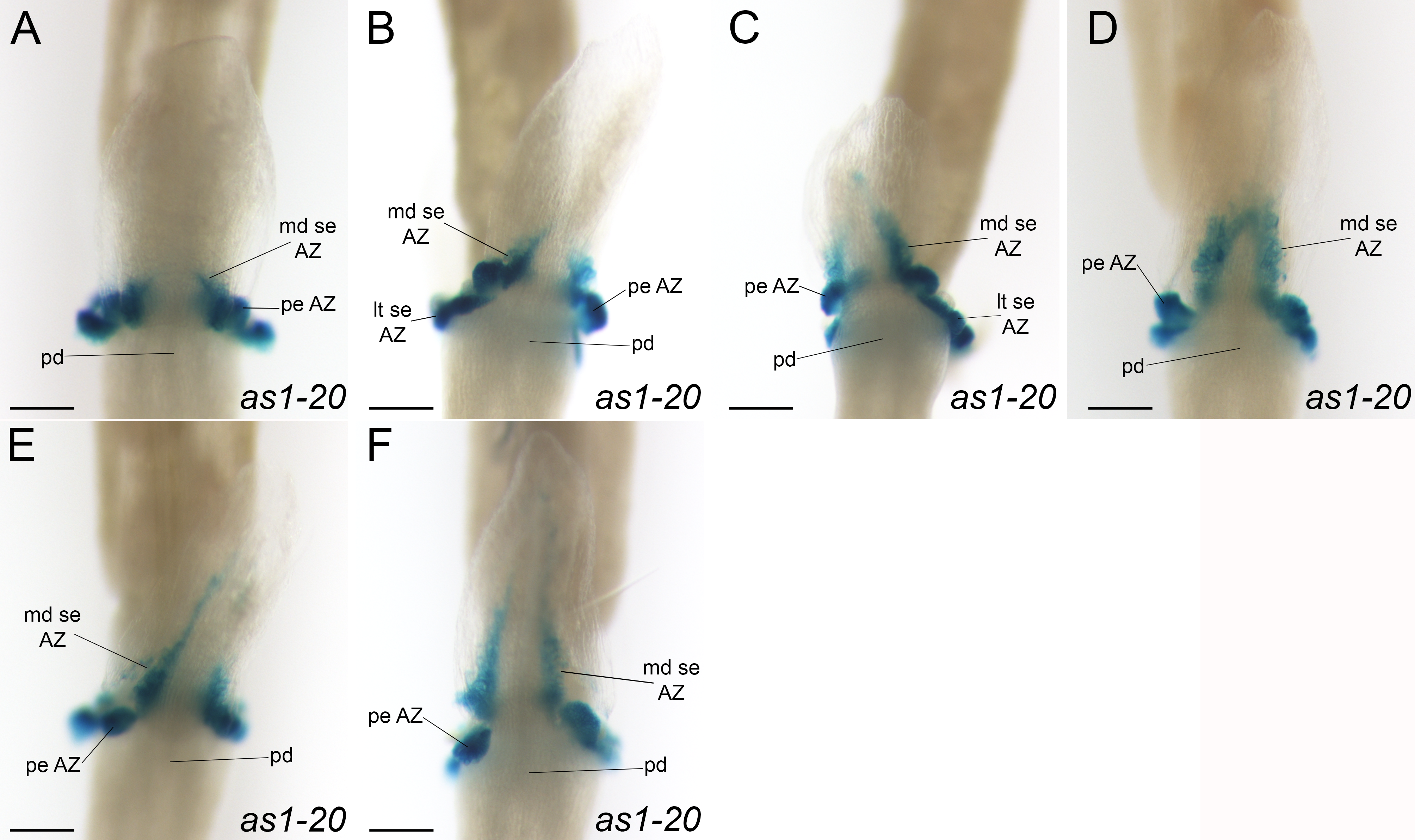

Supplement: Additional file 5: Figure S3. — Expression of the HAE::GUS marker progresses distally from the proximal edges of as1 medial sepals. Medial views of as1-20 mutant flowers (stage 17) histochemically stained for β-Glucuronidase (GUS) activity. Expression of GUS initiates at the proximal margins of the medial sepals (A, B). Stripes of GUS expression extend from each origin in a distal direction (B, C) until they intersect (D) to outline the edges of an inverted V-shaped proximal domain. In some as1-20 medial sepals (E, F), stripes of GUS expression expand distally toward the sepal tip without intersecting in a proximal region. [file s12870-014-0195-5-S5.tiff]
